# Fenretinide Causes Emphysema, Which Is Prevented by Sphingosine 1-Phoshate

**DOI:** 10.1371/journal.pone.0053927

**Published:** 2013-01-11

**Authors:** Masanori Yasuo, Shiro Mizuno, Jeremy Allegood, Donatas Kraskauskas, Harm J. Bogaard, Sarah Spiegel, Norbert F. Voelkel

**Affiliations:** 1 Pulmonary and Critical Care Medicine Division and Victoria Johnson Center for Obstructive Lung Diseases, Virginia Commonwealth University, Richmond, Virginia, United States of America; 2 First Department of Internal Medicine, Shinshu University School of Medicine, Matsumoto, Japan; 3 Department of Biochemistry, Virginia Commonwealth University, Richmond, Virginia, United States of America; University of Illinois College of Medicine, United States of America

## Abstract

Sphingolipids play a role in the development of emphysema and ceramide levels are increased in experimental models of emphysema; however, the mechanisms of ceramide-related pulmonary emphysema are not fully understood. Here we examine mechanisms of ceramide-induced pulmonary emphysema. Male Sprague-Dawley rats were treated with fenretinide (20 mg/kg BW), a synthetic derivative of retinoic acid that causes the formation of ceramide, and we postulated that the effects of fenretinide could be offset by administering sphingosine 1-phosphate (S1P) (100 µg/kg BW). Lung tissues were analyzed and mean alveolar airspace area, total length of the alveolar perimeter and the number of caspase-3 positive cells were measured. Hypoxia-inducible factor alpha (HIF-1α), vascular endothelial growth factor (VEGF) and other related proteins were analyzed by Western blot analysis. Immunohistochemical analysis of HIF-1α was also performed. Ceramide, dihydroceramide, S1P, and dihydro-S1P were measured by mass spectrometer. Chronic intraperitoneal injection of fenretinide increased the alveolar airspace surface area and increased the number of caspase-3 positive cells in rat lungs. Fenretinide also suppressed HIF-1α and VEGF protein expression in rat lungs. Concomitant injection of S1P prevented the decrease in the expression of HIF-1α, VEGF, histone deacetylase 2 (HDAC2), and nuclear factor (erythroid-derived 2)-like 2 (Nrf2) protein expression in the lungs. S1P injection also increased phosphorylated sphingosine kinase 1. Dihydroceramide was significantly increased by fenretinide injection and S1P treatment prevented the increase in dihydroceramide levels in rat lungs. These data support the concept that increased *de novo* ceramide production causes alveolar septal cell apoptosis and causes emphysema via suppressing HIF-1α. Concomitant treatment with S1P normalizes the ceramide-S1P balance in the rat lungs and increases HIF-1α protein expression via activation of sphingosine kinase 1; as a consequence, S1P salvages fenretinide induced emphysema in rat lungs.

## Introduction

Chronic obstructive pulmonary disease (COPD), and particularly emphysema, is a major and increasingly recognized global health problem and chronic lung tissue destruction determines a large component of the pathogenesis and morbidity of patients with this disease [1], [2]. Although chronic inflammation has been identified as an important finding and documented histologically [3]–[5], the cellular and molecular details of lung tissue destruction are still incompletely understood [6], [7].

Vascular endothelial growth factor (VEGF) has been proposed to be an integral part of the homeostatic adult lung structure maintenance program [8] and the expression of the VEGF ligand and the VEGF receptor 2 (VEGFR2 (KDR)) proteins has been shown to be decreased in human lung tissue and airway samples from patients with severe COPD/emphysema [9], [10]. The finding that the VEGF receptor blocker SU5416 induces pulmonary emphysema has been reported from our laboratory [11] and Petrache et al showed that SU5416 caused emphysema in mice [12] is associated with pulmonary ceramide generation [12]. Thus, mechanistically this SU5416-induced emphysema model can be explained by VEGF receptor blockade- related lung cell apoptosis and oxidative stress [13] driven by intracellular ceramide [12]. Consistent with this concept, Diab et al. also reported that stimulation of sphingosine 1-phosphate (S1P) signaling prevented the SU5416 VEGF receptor blockade-induced emphysema in mice [14], hypothesizing that a homeostatic balance between ceramide and S1P was disturbed by VEGF receptor blockade, and that S1P can restore this disturbed balance [14].

S1P is a highly bioactive sphingolipid metabolite involved in many cellular processes including proliferation, survival, and migration, as well as tissue responses such as angiogenesis and responses to allergens [15]. Whereas the role of S1P in the pathogenesis of asthma has been investigated [16], its contribution to the pathogenesis of COPD/emphysema is poorly understood [17].

S1P can activate HIF-1α in vascular cells [18]. We had recently demonstrated reduced HIF-1α protein expression in lungs from COPD patients [19]. Therefore, we hypothesized that S1P may induce HIF-1α in the lung tissue and may induce HIF-1α target genes and proteins, and thus may protect the lung against emphysema development. Here, we use fenretinide, an intracellular ceramide inducer, to generate emphysematous changes in the rat lung and examine whether fenretinide would cause emphysema by increasing ceramide production. We examined whether fenretinide-induced airspace enlargement was associated with a reduction of lung tissue HIF-1α and investigated whether S1P could restore the tissue expression of HIF-1α and prevent fenretinide-induced airspace enlargement.

## Materials and Methods

### Chemicals

Chemicals and materials were obtained from the following sources: S1P was from Cayman chemical (Ann Arbor, MI), Fenretinide was from Tocris bioscience (Ellisville, MO), the ECL system (Western Lightening® and Western Lightening® plus-ECL) was from PerkinElmer (Waltham, MA); NE-PER® Nuclear and Cytoplasmic Extraction Reagents were from Thermo Scientific (Rockford, IL); 4–12% Bis-Tris Nupage gels, and MES-SDS running buffer were from Invitrogen (Carlsbad, CA); the polyvinylidene difluoride (PVDF) membranes was from Bio-Rad Laboratories (Richmond, CA); the protease inhibitor cocktail was from Roche Applied Science (Indianapolis, IN); positive control of HIF-1α protein, rabbit anti-VEGF polyclonal antibody, mouse anti-Akt monoclonal antibody, rabbit anti-phospho Akt (pAkt) polyclonal antibody, rabbit anti-Nrf2 polyclonal antibody, mouse anti-HIF-1α monoclonal antibody, rabbit anti-HDAC2 polyclonal antibody, goat anti-Lamin B polyclonal antibody, and horseradish peroxidase-conjugated goat anti-mouse and rabbit, and donkey anti-goat IgG were from Santa Cruz Biotechnology Inc. (Santa Cruz, CA). Rabbit anti-active Caspase-3 antibody was from Cell Signaling Technology Inc. (Danvers, MA). Rabbit anti-phospho specific sphingosine kinase 1 antibody and rabbit anti-sphingosine kinase 1 polyclonal antibody were from ECM biosciences (Versailles, KY). Vectastain® Elite ABC-Peroxidase Kits Universal was from Vector Laboratories (Burlingame, CA). Liquid diaminobenzidine (DAB) substrate chromogen system was from Dako North America Inc. (Carpinteria, CA). All other chemicals were purchased from Sigma (St. Louis, MO).

#### Animal Experimental Protocols

The study was carried out in strict accordance with the recommendations published in the Guide for the Care and Use of Laboratory Animals of the National Institutes of the Health Guidelines for the Care and Use of Laboratory Animals (IACUC) and approved by the Virginia Commonwealth University’s Institutional Animal Care and Use Committee (Protocol Number: AM10162 Pathobiology of Emphysema). Fenretinide was dissolved in 1∶1:6 of ethanol, Cremophor® (Sigma) and normal saline. S1P was dissolved in 3% fatty acid free bovine serum albumin in phosphate buffered saline (PBS). Adult male Sprague-Dawley rats (200 g) were injected intraperitoneally with 20 mg/kg body weight of fenretinide two times per week for 4 weeks. S1P was injected intraperitoneally (100 µg/kg body weight) five days per week for 4 weeks, starting on day 1 of the fenretinide injections. The dosing and treatment schedule of fenretinide and S1P injection was based on previously published reports [20], [21]. Control rats received only solvents ((1∶1:6 of ethanol, Cremophor®, normal saline) and 3% fatty acid free bovine serum albumin (Sigma)) in PBS. For the entire study we used the following numbers of rats: control (n = 6), fenretinide (n = 5), S1P (n = 5), and fenretinide and S1P (n = 4). All surgery was performed under sodium pentobarbital anesthesia, and every effort was made to minimize pain and distress.

#### Morphometry

Lungs were inflated with 0.5% low-melting agarose at a constant pressure of 25 cm H_2_O, fixed in 10% formalin for 48 hours and paraffin-embedded by standard techniques. Sections (5 µm) were stained with hematoxylin and eosin. Images were acquired with a Carl Zeiss AxioCam color camera (Carl Zeiss Vision GmbH, Hallbergmoos, Germany) and analyzed using AxioVision® Imaging System software (Carl Zeiss Vision GmbH). 10 random lung fields per tissue section were captured at a 100× magnification, and then AxioVision® Imaging System software was used to measure the mean alveolar airspace areas (MAA) and total length of alveolar perimeters (TLAP) in pixels per µm^2^.

#### Western blot analysis

Cytoplasmic and nuclear proteins from lungs were prepared using NE-PER Nuclear and Cytoplasmic Extraction Reagents based on the manufacturer’s protocol (Pierce, Rockford, IL), and the protein extracts were analyzed for protein content using a Bradford protein assay [22]. Each sample was quantified, and then 40 µg of protein (cytoplasmic protein) or 20 µg of protein (nuclear protein) was loaded into each lane of a 4–12% Bis-Tris Nupage gel with MES SDS running buffer, according to the manufacturer’s protocol. The gel was transferred to a PVDF membrane by electrophoresis at 100 V for 1 to 1.5 hour. The membrane was blocked in PBS, 0.2% Tween 20 (PBS-T), and 5% nonfat milk at room temperature for 1 hour. All antibodies were diluted in the same blocking buffer. The membrane was then probed with the primary antibodies. Subsequently, membranes were incubated with horseradish peroxidase-conjugated goat anti-mouse, goat anti-rabbit, or donkey anti-goat antibody. The ECL system was used for detection of the proteins.

#### Immunohistochemical staining for active Caspase-3 and HIF-1α

The slides with 5 µm paraffin sections were deparaffinized in xylene, rehydrated by serial immersions in 100% ethanol, 95% ethanol, 70% ethanol, and then washed with PBS. Then the sections were rehydrated and submitted to microwave treatment (800 W/15 min) in 10 mM citric acid monohydrate solution, and then quenching of endogenous peroxidase with 3% H_2_O_2_ for 15 minutes. Sections were incubated overnight with anti-cleaved caspase-3 rabbit polyclonal antibody (1∶200 dilution) or anti- HIF-1α-mouse monoclonal antibody (1∶10 dilution) at 4°C and then were incubated with rabbit (for active caspase-3) or mouse (for HIF-1α)-labeled polymer horseradish (HRP) peroxidase for 30 min at room temperature. Following the secondary antibody application, sections were incubated with ABC complex (Vector, Burlingame, CA) for 30 min at room temperature, rinsed in PBS, and developed with diaminobenzidine (DAB; Vector, Burlingame, CA) and hydrogen peroxide. Washing with water stopped the DAB reaction. A light hematoxylin counterstain was applied. Sections were dehydrated by sequential immersion in 70% ethanol, 95% ethanol, 100% ethanol, 95% ethanol and then xylene before placing a coverslip on the section. For a negative control, the primary antibody was omitted.

#### Apoptotic index

After immunohistochemical staining of active caspase-3, 10 random lung fields per tissue section were captured at a 400× magnification, then the number of active caspase 3-positive cells was counted by an observer who was blinded to the coded tissue sections using the AxioVision® Imaging System software. The total length of the alveolar perimeters of each of the captured images was used as the reference of the caspase-3 positive index.

#### Quantification of HIF-1α positive cells

The quantification of HIF-1α positive cells was previously reported [19]. Briefly, after immunohistochemical stain of HIF-1α, images were acquired and analyzed using AxioVision® Imaging System software (Carl Zeiss Vision GmbH). 10 random lung fields per slide were captured at a 400× magnification, then the AxioVision® Imaging System software was used to measure the total length of the alveolar perimeters in pixels per µm^2^, and then the number of HIF-1α positive cells was counted by an observer who was blinded to the coded tissue sections.

#### Mass spectrometric analysis of sphingolipid metabolites

Fresh frozen (−80°C) rat lung tissue was used for measuring sphingolipid metabolites. After homogenization, an internal standard cocktail was added (0.5 nmol each C12∶0-SM, C12∶0-Cer, C12∶0-GlcCer, d17-sphingosine, d17-sphinganine, d17-sphingosine-1-phosphate, d17-sphinganine-1-phosphate, and C12∶0-Cer-1-phosphate from Avanti Polar Lipids, Alabaster, AL), lipids extracted, and individual ceramide acyl chain species quantified after liquid chromatography, the electrospray ionization-tandem mass spectrometry (LC-ESI-MS/MS, 4000 QTRAP; Applied Biosystems, Foster City, CA) as described previously [23]. Data are expressed as pmoles per mg of tissue.

#### Statistical analysis

All data are expressed as means ± standard error (S.E.M.). Data were evaluated for significance by an ANOVA and the Tukey or the Kruskall-Wallis test. Significance was determined at *P*<0.05 (two-tailed test).

## Results

### Chronic Administration of Fenretinide Induces Emphysema in Rat Lung

**Figure 1:**
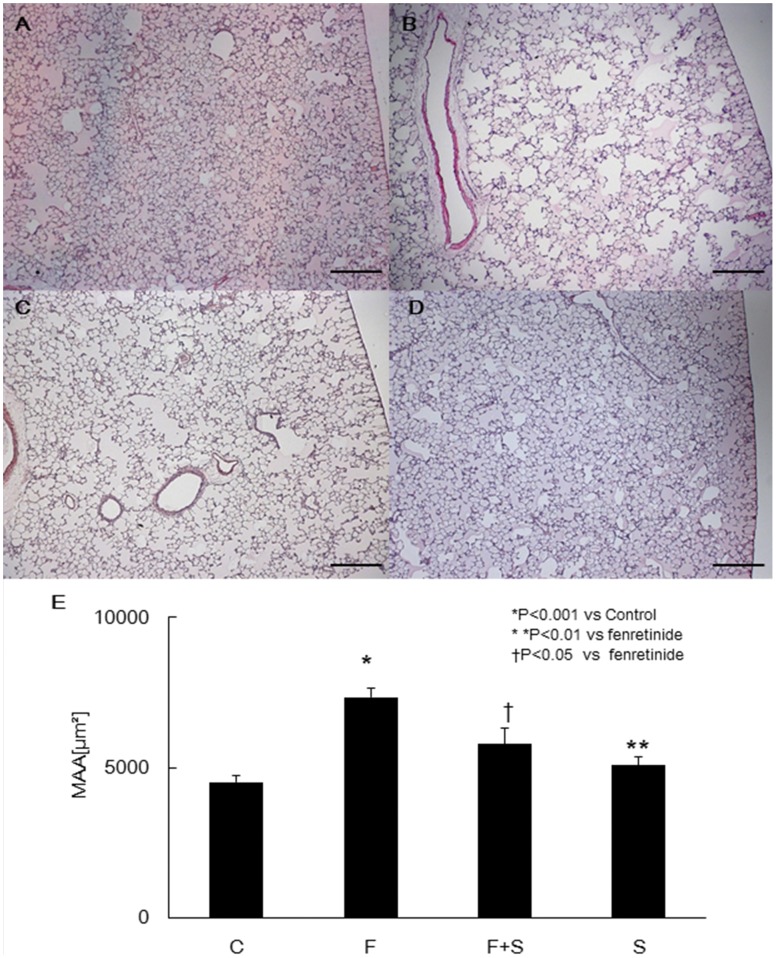
Morphological analysis of the lungs from fenretinide challenged rats treated with or without sphingosine 1-phosphate (S1P). When compared to control lungs (A), the low power magnification shows air-space enlargement in the chronic fenretinide treated rat lungs (B). Examples of data based on concurrent S1P administration and S1P alone treatment are shown in (C) and (D), respectively. Quantitative analysis is shown in (E). Data are expressed as mean ± SEM. C = Control, F = Fenretinide, S = S1P Bars = 250 µm, Original Magnification x40.

Following four weeks of fenretinide injections, we harvested and examined the lungs and performed morphometrical analysis. When compared to control rat lungs, fenretinide treated rats demonstrated emphysema like airspace enlargement (Fig. 1A and B). Concurrent S1P administration prevented the air-space enlargement (Fig. 1C). No morphological changes were observed after S1P treatment (Fig. 1D). The mean alveolar airspace areas (MAA) were significantly larger than in control rats (Fig. 1E).

### Fenretinide Increases Lung *de novo* Synthesis of Ceramide

**Figure 2:**
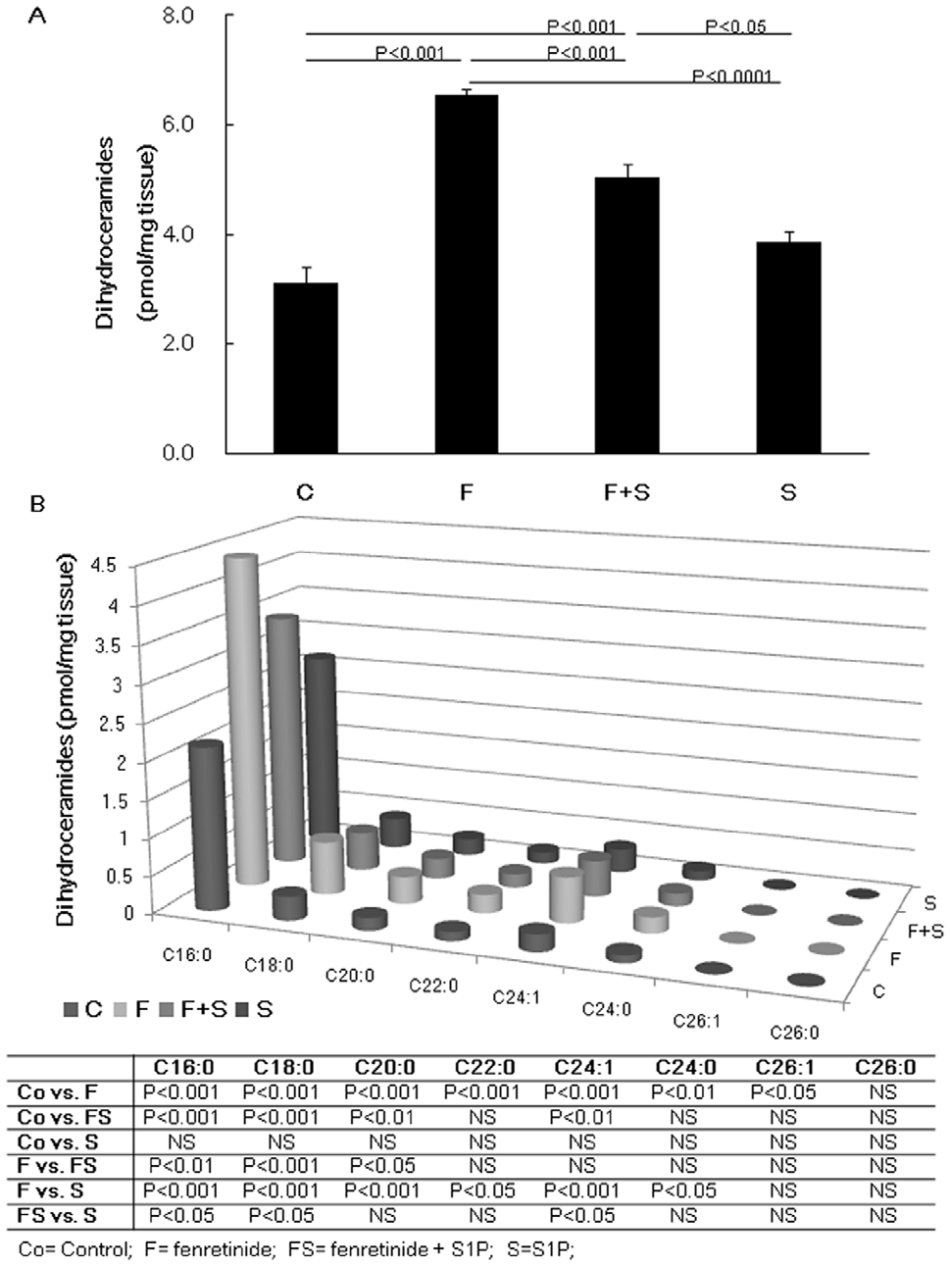
Mass spectrometric analysis of dihydroceramide and long chain ceramide species. The concentrations of dihydroceramide in the rat lungs are shown (A). The individual data of the dihydroceramide species are depicted graphically and numerically (B). Data are expressed as mean ± SEM. C = Control, F = Fenretinide, S = S1P.

Sphingolipids were extracted and analyzed by LC-ESI-MS/MS [23]. In our study, chronic fenretinide treated rat lungs showed significantly higher levels of dihydroceramide than the control rat lungs (Figure 2A). We also analyzed long chain species of dihydroceramide and as indicated in Figure 2B, the main component of the lung tissue extract dihydroceramide was C16∶0. The results of the statistical analysis for each ceramide species are also displayed in Figure 2B. In contrast, fenretinide did not significantly change the ceramide concentration in rat lungs (data not shown). These results agree with recent studies demonstrating that fenretinide induces dihydroceramide formation via both serine palmitoyltransferase and dihydroceramide synthase [24] while concomitantly inhibiting dihydroceramide desaturase [25], [26], suggesting that dihydroceramide rather than ceramide mediates the fenretinide-induced effects.

### Chronic Injection of Fenretinide Induces Lung Cell Apoptosis

**Figure 3:**
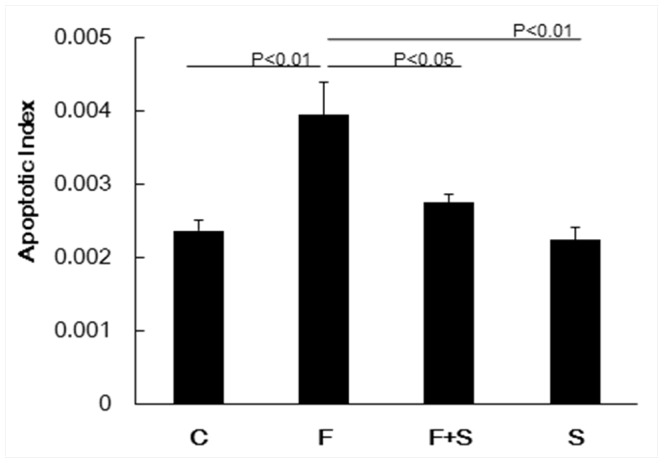
Apoptotic index of rats from animals with fenretinide with or without S1P. Immunohistochemical staining for cleaved caspase-3 was performed and used to calculate the apoptotic index. After immunohistochemical staining, 10 random lung fields per tissue section were captured at a 400× magnification, then the number of active caspase 3-positive cells was counted by an observer who was blinded to the coded tissue sections using the AxioVision® Imaging System software. The total length of the alveolar perimeters of each of the captured images was used as reference for the caspase-3 positive index. Data are expressed as mean ± SEM. C = Control, F = Fenretinide, S = S1P.

Immunohistochemical staining for cleaved caspase-3 was performed and we calculated the apoptotic index. When compared to control rats, fenretinide treatment resulted in the generation of a significantly larger number of cleaved caspase-3 positive cells in the lungs (Figure 3).

### Systemic Administration of S1P Reduces Dihydroceramide Levels and Improves Fenretinide Induced Airspace Enlargement and Lung Cell Apoptosis

As indicated in Figures 2A and B, intraperitoneal administration of S1P significantly reduced the dihydroceramide levels in fenretinide treated rat lungs. S1P administration also reduced fenretinide-induced lung cell apoptosis (Figure 3) and improved the emphysema like airspace enlargement of the rat lung (Figure 1A to E).

### Fenretinide Disrupts the Signaling of Cell Survival Factors and Administration of S1P Prevents this Disruption

**Figure 4:**
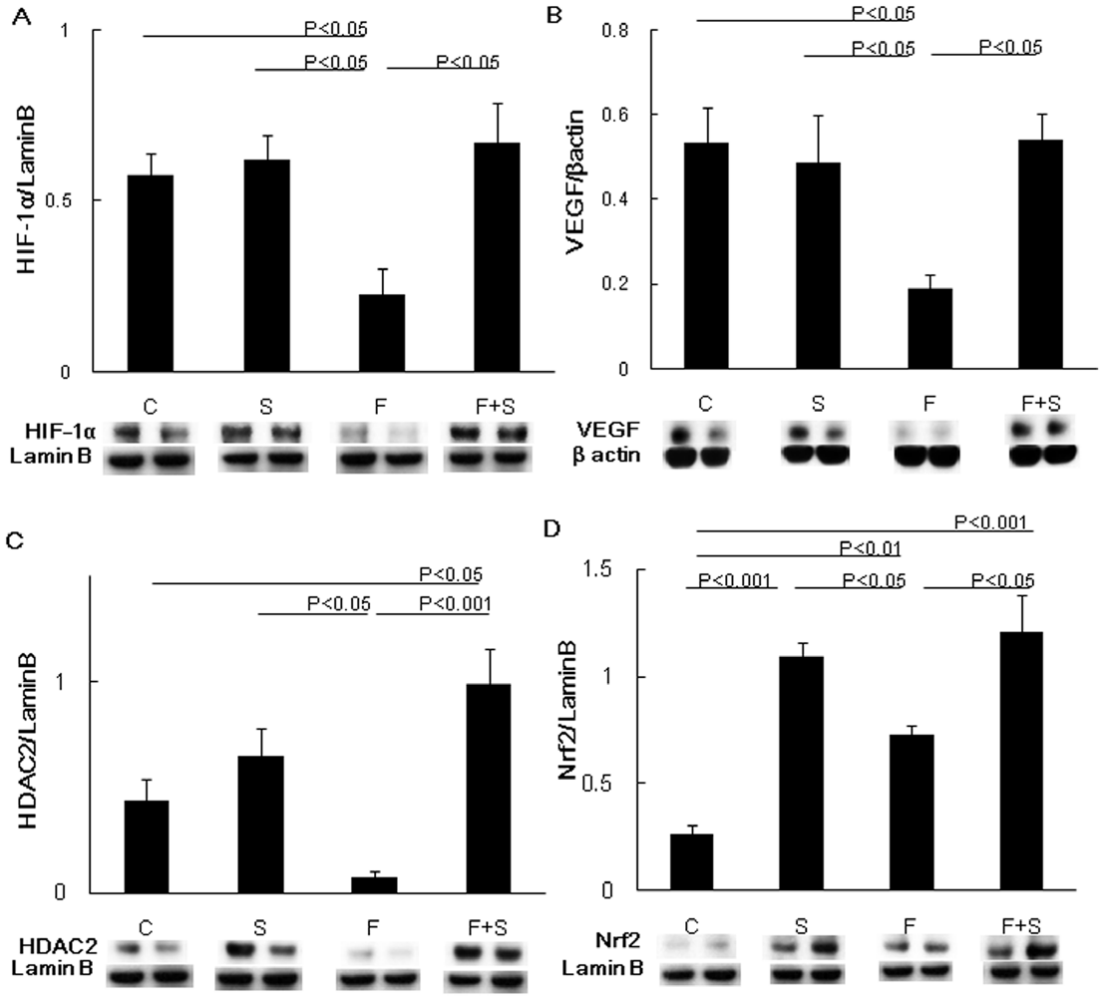
Western blot analysis of cytoplasmic and nuclear proteins (n = 4, for each group). Densitometric data and representative protein bands are shown. HIF-1α, HDAC2 and Nrf2 expression was normalized to a housekeeping protein Lamin B (A, C and D, respectively). VEGF expression was normalized to β actin (B). Data are expressed as mean ± SEM. C = Control, F = Fenretinide, S = S1P.

Having reported that HIF-1α and VEGF operate as lung structure maintenance factors [9], [11], [13], [19], in particular that HIF-1α protein expression was suppressed in the lungs from patients with COPD/emphysema, we next investigated whether the expression of HIF-1α and VEGF was affected by fenretinide treatment. Western blot analysis showed significantly reduced HIF-1α and VEGF protein expression (Figure 4A and B) as a consequence of chronic fenretinide treatment. HDAC2 and the transcription factor Nrf2, known to induce a number of antioxidant genes, were similarly reduced in expression by chronic fenretinide treatment (Figure 4C and D).

**Figure 5:**
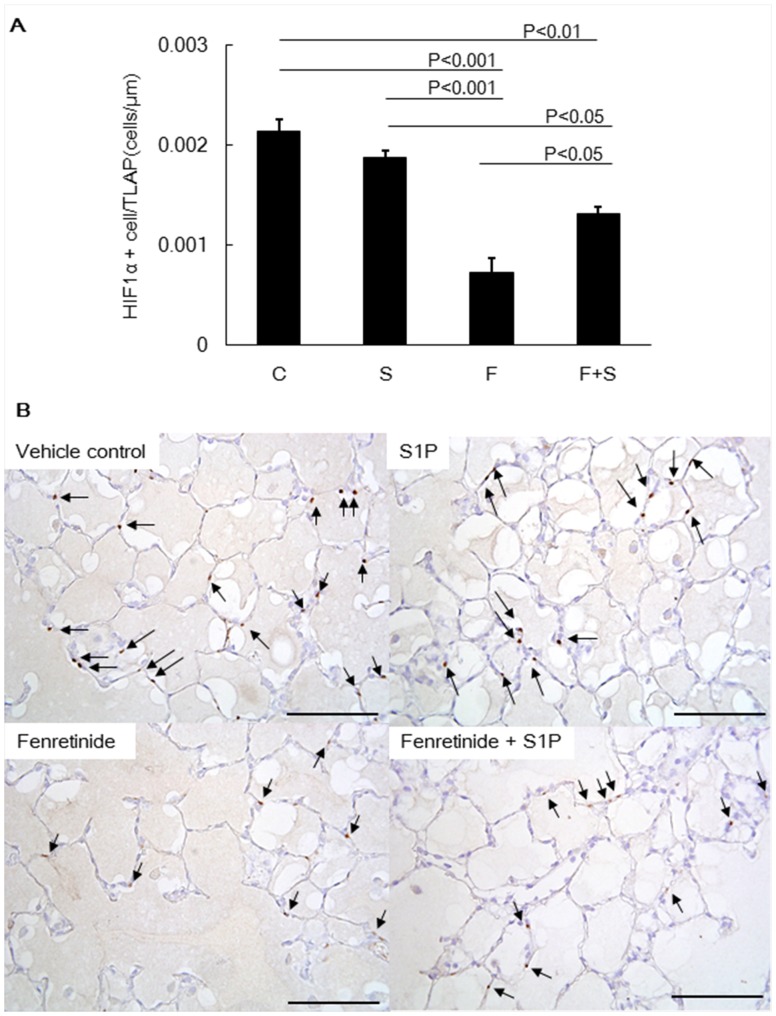
Immunohistochemical analysis of HIF-1α. The number of the HIF-1α positive cells is counted in vehicle control, S1P, fenretinide, and fenretinide with S1P treated rat lungs. Then they were referenced to the total length of the alveolar perimeters (A). Representative images of immunohistochemical staining of HIF-1α are shown (B). Bars = 50 µm, Original Magnification x100 Data are expressed as mean ± SEM. C = Control, F = Fenretinide, S = S1P, TLAP =  total length of the alveolar perimeters.

Because HIF-1α protein expression in lung is considered a key regulator of air space homeostasis, we examined HIF-1α using immunohistochemical analysis as described previously [19]. A significantly reduced number of HIF-1α positive cells were seen in fenretinide treated rat lungs (Figure 5A). S1P treatment alone did not change the expression of HIF-1α (Figure 4A and 5A, B). Representative immunohistochemistry is shown in Figure 5B.

Concomitant S1P administration protected the lungs against these fenretinide-triggered lung tissue protein expression changes. (Figure 4A to D and 5A, B).

### Administration of S1P Induces Phospho-sphingosine Kinase 1 and Reverses the Ceramide-S1P Imbalance

**Figure 6:**
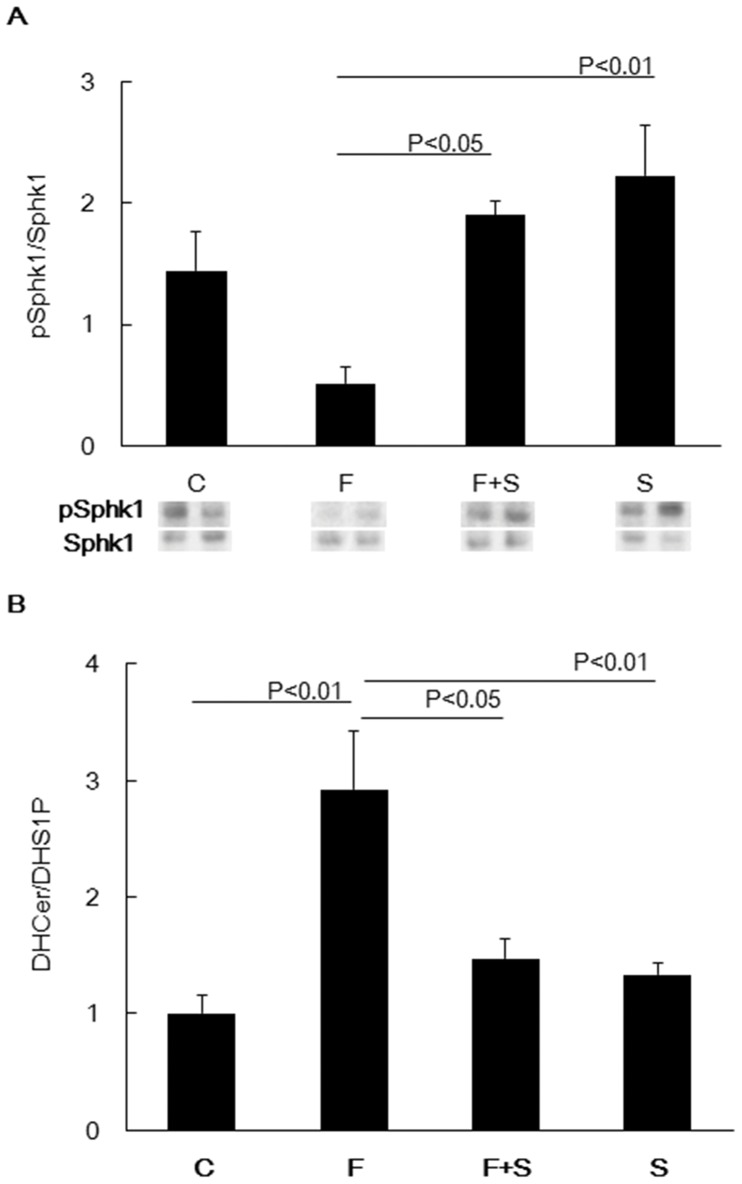
Effect of exogenous S1P on phospho-sphingosine kinase 1 (pSphk1) and the balance of dihydroceramide and dihydro-S1P (n = 4, each for group). Densitometric data and representative protein bands are shown. Phospho-Sphk1 was adjusted to Sphk1 (A). The balance of dihydroceramide/dihydro-S1P was investigated by mass spectrometric analysis (B). Data are expressed as mean ± SEM. C = Control, F = Fenretinide, S = S1P.

It has been reported that exogenously administered S1P activates sphingosine kinase 1 and induces dihydro-S1P formation [27]. We therefore investigated whether lung tissue sphingosine kinase 1 was activated following intraperitoneal S1P injection in fenretinide treated rats. When compared to the lungs from fenretinide treated rats, injection of fenretinide plus S1P increased the expression of phosphorylated sphingosine kinase 1 in the lung tissues (Figure 6A). When compared to the lungs from S1P alone treated and control rats, exogenous administrations of S1P protected against the effects of fenretinide treatment (Figure 6A). Because fenretinide induces dihydroceramide (Figure 2) and administration of S1P induces dihydro-S1P [25], [28], we questioned whether there was an effect of S1P treatment on the balance between dihydroceramide and dihydro-S1P. Mass spectrometric analysis of dihydroceramide and dihydro-S1P revealed that there was a significant increase of the dihydroceramide/dihydro-S1P ratio in lung tissues from fenretinide treated rats and that this ratio was normalized by the administration of S1P (Figure 6B).

## Discussion

In this study, we have shown that chronic injection of fenretinide induced ceramide generation in rat lungs. The dihydroceramide production disrupted the homeostatic balance between dihydroceramide and dihydro-S1P, resulting in a reduction of HIF-1α, and VEGF expression, and lung cell apoptosis, and alveolar airspace enlargement. Exogenously administered S1P activated Sphk1 leading to intracellular production of S1P. Thus increased S1P restored the S1P/ceramide rheostat, the expression of HIF-1α and VEGF and prevented lung cell apoptosis. As a result, the airspace enlargement was prevented.

Although increased ceramide levels in emphysematous lung tissue have been associated with induction of lung cell apoptosis, little is known about the role of dihydroceramide, a *de novo* pathway ceramide precursor. To begin to examine this pathway we utilized fenretinide (4-hydroxyphenylretinamide, 4HPR) an anti-cancer drug which is currently undergoing clinical trials [20], [21]. Fenretinide has been found to affect sphingolipid metabolism and to increase dihydroceramide levels. Fenretinide increases dihydroceramide and synergizes with dimethylsphingosine to enhance cancer cell killing [25], [26].

Here we expand on the body of work that connects oxidative stress with ceramide and emphysematous lung destruction [12], [14] by demonstrating that chronic treatment of rats with fenretinide, a synthetic retinoid, presently in use as an apoptosis-inducing cancer therapeutic agent [29], causes emphysematous airspace enlargement. This is to our knowledge the first report of experimentally induced lung tissue destruction by fenretinide. It has been reported that ceramides induce lung endothelial cell and epithelial cell apoptosis [28], [30], and we thus examined whether chronic fenretinide treatment caused lung cell apoptosis. Because fenretinide causes activation of protein kinase C (PKC) [31] and induction of the transcription factor GADD153 [32], which in turn have been associated with fenretinide-induced apoptosis, we examined the expression of cleaved caspase 3 in lungs from fenretinide treated rats and demonstrated the presence of a large number of lung structure cells undergoing apoptosis. We therefore conclude that fenretinide-induced airspace enlargement is due to lung cell apoptosis.

These results led us to further examine the actions of this retinoid in the lung in the context of the homeostatic sphingosine-1-phosphate (S1P)/ceramide balance as previously described by Takabe et al [15] and by Huwiller and Pfeilschiffer [33]. S1P acts on intracellular targets, but S1P is also released from cells and acts on cell surface S1P receptors in an autocrine or paracrine fashion [17]. Whereas S1P is proangiogenic and suppresses apoptosis, ceramide induces apoptosis. Together Sphingosine, S1P and ceramide represent a rheostat which determines cell fate [34].

Whereas Petrache et al [12] showed that one consequence of VEGF receptor blockade was ceramide production in the lung tissue, we stimulated ceramide production by systemically administering fenretinide. Our finding that fenretinide caused an increase in the tissue levels of dihydroceramide indicates activation of the endoplasmic reticulum-localized *de novo* biosynthetic pathway of sphingolipids [35]; the most abundant dihydroceramide species produced in the lung by fenretinide was C16∶0 dihydroceramide (Figure 2B).

We also showed that exogenously administered S1P did protect against fenretinide-induced lung cell apoptosis (Figure 3) and emphysema (Figure 1). This is surprising because intraperitoneal injection of S1P- which has a short biological half-life -was effective in neutralizing the ceramide actions in the lung. The protective effect of injected S1P was associated with the preservation of the transcription factorHIF-1α expression (Figure 5) and increased Nrf2 expression. In addition, we also found that the reduction in the expression of HDAC2 was prevented by S1P. The HDAC expression reduction was accompanied by a decrease in the expression of the HIF-1α and VEGF (Figure 4). As we have previously reported [11], [36], [37], inflammation was assessed histologically in this model, i.e., multiple tissue sections were examined, and inflammatory cell infiltrates and accumulations of alveolar macrophages or neutrophils were not found in the lung parenchyma or immediate peribronchial or perivascular areas. At least in rats emphysema associated with decreased expression of HDAC2, HIF-1α and VEGF is not due to inflammation. However, our data recapitulate findings obtained after the examination of lung tissue extracts from patients with COPD: reduced VEGF [9] and HDAC2 protein [19] expression. In the lung tissue samples from COPD patients diminished expression of the gene controlling the expression of a number of antioxidant enzymes, Nrf2, has previously been reported [38], [39]. However, in the fenretinide treated rat lungs, we observed increased Nrf2 protein expression perhaps reflecting an attempt of the tissue to counter ceramide-related oxidant stress. Thus, with the exception of the Nrf2 expression, the pattern of reduced protein expression found in COPD lungs was also detected in the lungs from fenretinide treated emphysematous animals (Figure 4).

There are a number of known interactions between HIF-1α and sphingolipids [18], [40], [41] and also between S1P and HDAC [42]. For example HIF-1α is a sphingosine kinase 1 regulated gene [42], [43] and HDACs appear to be intracellular targets of S1P [43], [44]. That fenretinide can alter HDAC expression has not been previously reported (Figure 4C).

It is known that exogenous S1P activates sphingosine kinase 1 and increases intracellular S1P [27], and here we show that i.p. injection of S1P increased the amount of phosphorylated sphingosine kinase 1 in the lung tissue from chronically fenretinide treated rats (Figure 6A). In addition, S1P normalized the dihydroceramide/dihydro-S1P ratio in the lung tissue (Figure 6B).

**Figure 7:**
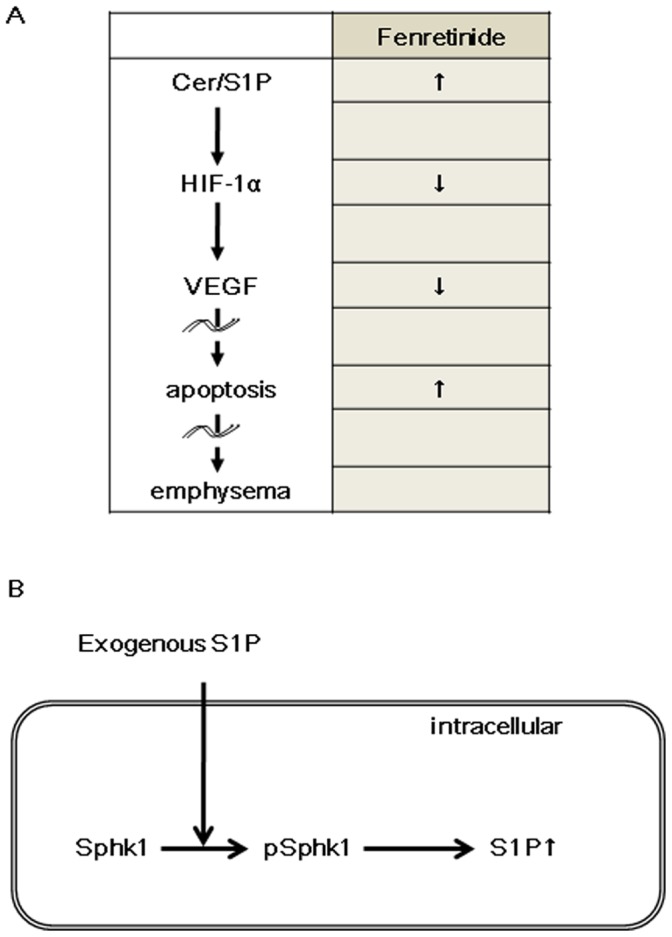
The schematic represents the concept of how fenretinide and exogenously administered sphingosine 1 phosphate (S1P) affect the adult lung structure maintenance. Administration of fenretinide changes the ceramide/S1P ratio by increasing the generation of ceramide, which in turn decreases HIF-1α and VEGF expression in the lung. Reduction of HIF-1alpha and VEGF -both central to the adult lung structure maintenance program- causes emphysema (A). Exogenously administered S1P increases the amount of intracellular S1P via sphingosine kinase 1 (Sphk1) activation (B), thus protecting against lung cell apoptosis.

Taken together, our experimental data support the concept that fenretinide, via dihydroceramide, causes airspace enlargement which can be prevented by exogenous S1P (Figure 1A–E), which in turn maintains HIF-1α protein expression and the expression of the HIF-1α target gene VEGF (Figure 4 A and B). We speculate that fenretinide induces endothelial cell apoptosis (Figure 3, 7A) and that lung endothelial cell-derived S1P “buffers” the action of ceramides generated after fenretinide treatment (Figure 7B). It has previously been shown that fenretinide causes endothelial cell apoptosis and it has been suggested that ceramide signals upstream from caspases [44], [45]. Fenretinide has been shown to inhibit tube formation in angiogenesis assays [46] and dihydroceramide likely is responsible for the antiangiogenic action of fenretinide. In our experiments, fenretinide treatment caused a reduction in the lung tissue HIF-1α protein which was prevented by concomitant S1P treatment (Figure 4A, 5A, B), while S1P treatment increased Nrf2 protein expression (Figure 4D). We propose that the administration of exogenous S1P has induced intracellular S1P via sphingosine kinase 1 activation (Figure 6A). Whether the protective/preventative action of administered S1P during fenretinide challenge of the animals was due to the upregulation of HDAC2 (Figure 4C) or VEGF or Nrf2- or due to a combination of these effects, or other S1P controlled actions, for example, effects of S1P on lipoxygenases [47], remains unclear.

We have previously suggested that disruptions of the adult lung maintenance program causes emphysema [8], [48]. First, we described that the disruption of VEGF signaling causes lung cell apoptosis and alveolar air space enlargement [11]. In follow-up studies we showed that downregulation of HIF-1α by copper depletion [36] or HDAC inhibition [37] causes downregulation of VEGF expression, lung cell apoptosis, and alveolar enlargement. Apparently “too much or too little of VEGF is catastrophic to the lung” [49]. The homeostatic concept of the “adult lung cell maintenance program” can explain how the adult lung maintains its structural integrity. This concept is based on the postulate that there are factors which are of fundamental importance for the proper maintenance of the organ structure such as HIF-1α and VEGF [48]. We now propose to add the S1P/ceramide balance or “rheostat” to the repertoire of lung structure maintenance factors. An offset of the S1P/ceramide rheostat affects the expression of HDAC, HIF-1α and VEGF resulting in lung cell apoptosis (Figure 7A). Exogenous administration of S1P activates Sphk1 to generate intracellular S1P and resets the rheostat, thus preventing emphysematous development (Figure 7B).

In conclusion, our experiments support the concept that emphysematous lung tissue destruction can occur independent of inflammation via an imbalance of the S1P/ceramide homeostasis. This “sphingolipid rheostat” appears to influence HIF-1α-dependent VEGF transcription, and HDAC2 and Nrf2 expression. Each of these factors are components of the adult lung structure maintenance program [48].
